# Supplementary material for: Simultaneous tensile and shear measurement of the human cornea in vivo using S0- and A0-wave optical coherence elastography
Source: ArXiv. 2023 Aug 10:arXiv:2308.05316v1. Preprint. [Version 1] (PMC10441437)
Supplement: Supplement 1 [file NIHPP2308.05316v1-supplement-1.pdf]

Supplementary materials

Simultaneous tensile and shear measurement of the human cornea *in vivo* using S0- and A0-wave optical coherence elastography

Guo-Yang Li[1,†,#], Xu Feng[1,†], Seok-Hyun Yun[1,2,*]

[1] Harvard Medical School and Wellman Center for Photomedicine, Massachusetts General Hospital, 50 Blossom St., Boston, MA 02114, USA.

[2] Harvard-MIT Division of Health Sciences and Technology, Cambridge, MA 02139, USA.

[#] Current address: Department of Mechanical Engineering, Peking University, Peking, China

[†] Equal contribution

*Correspondence: syun@hms.harvard.edu

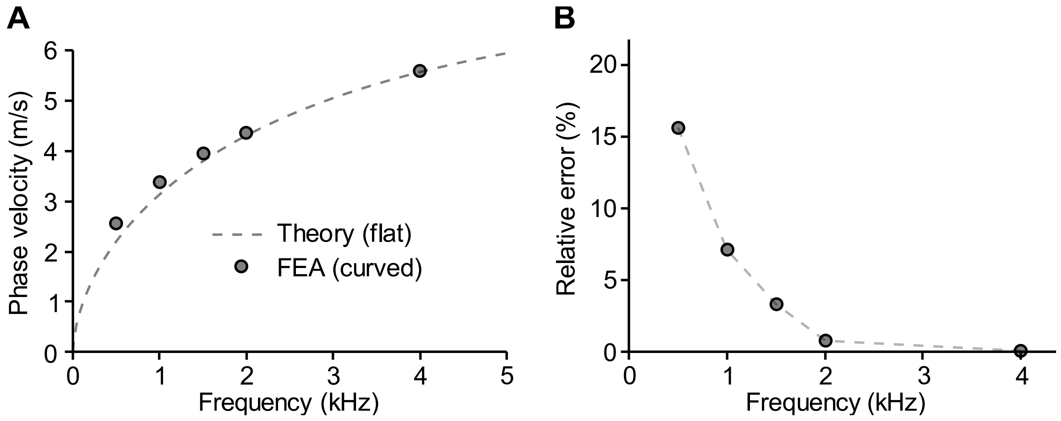

Fig. S1 Influence of corneal curvature on wave speed. (A) Phase velocity dispersion relation. Dashed line: theoretical model assuming the cornea is flat. Circles: Finite element analysis (FEA) simulation considering corneal curvature. (B) Relative error of the phase velocities between the theory for the flat cornea and the FEA simulation of the curved cornea. The relative error becomes negligible at 4 kHz, as the wavelength is significantly smaller than the radius of curvature.

Supplementary Note S1. Acoustoelastic model for guided waves in the cornea

The wave equation for small-amplitude plane elastic wave in a prestressed solid, which can be found in Ref. (*1*), is

$$\alpha\frac{\partial^4\psi}{\partial x_1^4}+2\beta\frac{\partial^4\psi}{\partial x_1^2\partial x_2^2}+\gamma\frac{\partial^4\psi}{\partial x_2^4}=\rho\left(\frac{\partial^4\psi}{\partial x_1^2\partial t^2}+\frac{\partial^4\psi}{\partial x_2^2\partial t^2}\right),\tag{S1}$$

where the stream function ψ is related to the displacement components u_1 and u_2 via the relation of $u_1=\psi_{,2}$ and $u_2=-\psi_{,1}$. These relations promise $u_{1,1}+u_{2,2}=0$, which is equivalent to the material incompressible constraint adopted in this study. ρ and t denote the density and time, respectively. The coefficients α, β, and γ are determined by the constitutive law and the stretch ratio λ,

$$\alpha=\mathcal{A}^0_{1212},2\beta=\mathcal{A}^0_{1111}+\mathcal{A}^0_{2222}-2\mathcal{A}^0_{1122}-2\mathcal{A}^0_{1221},\gamma=\mathcal{A}^0_{2121},\tag{S2}$$

where the fourth-order tensor, A_{0ijkl}, is the Eulerian elasticity tensor and is defined as

$$\mathcal{A}^0_{ijkl}=F_{iI}F_{kJ}\frac{\partial^2 W}{\partial F_{jI}\partial F_{lJ}},\qquad i,j,k,l,I,J\in\{1,2,3\}\tag{S3}$$

where $\mathbf{F}$ is the deformation gradient tensor and W is the strain energy function. In Eq. (S3) the Einstein summation convention is adopted. For the HGO model, the strain energy is

$$W=\frac{\mu}{2}(I_1-3)+\frac{k_1}{k_2}\sum_{i=1}^{2}\left\{e^{k_2[\kappa(I_1-3)+(1-3\kappa)(I_{4i}-1)]^2}-1\right\},\tag{S4}$$

where μ, k_1, k_2 and κ are constitutive parameters. μ denotes the initial shear modulus. The dimension of k_1 is the same as μ, whereas k_2 is a dimensionless parameter which determines the nonlinear hardening effect of the collagen fibrils when being stretched. $\kappa=0$ if the collagen fibrils are ideally aligned (2), which is appliable for the cornea. $I_1=\mathrm{tr}(\mathbf{F}^\mathrm{T}\mathbf{F})$. I_{41} and I_{42} are two invariants related to two families of collagen fibers. Following the coordinate system (x_1,x_2,x_3) shown in Fig. 1C, the axes of the collagen fibers of the cornea, denoted by unit vectors $\mathbf{M}$ and $\mathbf{M}'$, are aligned with x_1 and x_3, i.e., $\mathbf{M}=(1,0,0)^\mathrm{T}$ and $\mathbf{M}'=(0,0,1)^\mathrm{T}$. Then I_{41} and I_{42} can be determined by $\mathbf{M}$ and $\mathbf{M}'$ (*2*)

$$I_{41}=(\mathbf{FM})\cdot(\mathbf{FM}),\quad I_{42}=(\mathbf{FM}')\cdot(\mathbf{FM}').\tag{S5}$$

With the strain energy function and the deformation tensor, the Cauchy stress tensor can be determined by

$$\sigma_{ij}=F_{iI}\,\partial W/\partial F_{jI}-\bar{p}\delta_{ij},\tag{S6}$$

where $\bar{p}$ is a Lagrange multiplier for material incompressibility and δ_{ij} is the Kronecker delta.

In this study, we consider a biaxial stretch, $\mathbf{F}=\mathrm{diag}(\lambda,\lambda^{-2},\lambda)$, where λ is the stretch ratio along x_1 (or x_3) axis. With $\sigma_{22}=0$ we can obtain ($\sigma=\sigma_{11}=\sigma_{33}$)

$$\sigma=\mu(\lambda^2-\lambda^{-4})+2k_1\lambda^2(\lambda^2-1)e^{\left[k_2(\lambda^2-1)^2\right]}.\tag{S7}$$

According to the Young-Laplace equation, $\sigma=\mathrm{IOP}\times R/(2h)$. Therefore, the stretch ratio λ can be obtained by solving the nonlinear equation

$$\mu(\lambda^2-\lambda^{-4})+2k_1\lambda^2(\lambda^2-1)e^{\left[k_2(\lambda^2-1)^2\right]}=\text{IOP}\times R/(2h). \tag{S8}$$

The coefficients α, β, and γ can be obtained by inserting Eqs. (S3) and (S4) into Eq. (S2),

$$\begin{gathered}\alpha=\lambda^2\left\{\mu+2k_1(\lambda^2-1)e^{\left[k_2(\lambda^2-1)^2\right]}\right\},\qquad \gamma=\mu\lambda^{-4}.\\ 2\beta=\alpha+\gamma+4k_1\lambda^4[2k_2(\lambda^2-1)^2+1]e^{\left[k_2(\lambda^2-1)^2\right]},\end{gathered} \tag{S9}$$

In the absence of prestress (i.e., $\lambda=1$)

$$\alpha=\mu,\beta=\mu+2k_1,\gamma=\mu. \tag{S10}$$

Next, we consider the guided wave motion in the cornea. It should be noted the boundary conditions (BCs) have pronounced effects on the dispersion relations of the guided waves. The two sides of the cornea are air and aqueous humor. In our simplified model (see Fig. 1C), the aqueous humor is modeled as a semi-infinite fluid layer and the wave equation is

$$\nabla^2\chi=\frac{\rho^f}{\nu}\chi_{,tt}, \tag{S11}$$

where ν (2.2 GPa) and ρ^f (1,000 kg/m^3) denote the bulk modulus and density of the fluid, respectively. The potential function χ is related to the displacement of the fluid (denoted by $\mathbf{u}^f$) via the relations of $u_1^f=\chi_{,1}$ and $u_2^f=\chi_{,2}$. The pressure of the fluid, denoted by p^*, is determined by

$$p^*=-\nu\nabla\cdot\mathbf{u}^f. \tag{S12}$$

At the interreference ($x_2=0$), the following interfacial conditions apply

$$u_2=u_2^f,\quad -\gamma\psi_{,11}+\gamma\psi_{,22}=0,\quad \rho\psi_{,2tt}-(2\beta+\gamma)\psi_{,112}-\gamma\psi_{,222}=-p_{,1}^*. \tag{S13}$$

At $x_2=h$, the boundary is stress free, which gives

$$-\gamma\psi_{,11}+\gamma\psi_{,22}=0,\quad \rho\psi_{,2tt}-(2\beta+\gamma)\psi_{,112}-\gamma\psi_{,222}=0. \tag{S14}$$

More details on the derivations of the boundary conditions can be found in Ref. (*3, 4*).

We seek the plane wave solutions for $\psi(x_1,x_2,t)$ and $\chi(x_1,x_2,t)$, i.e.,

$$\begin{cases}\chi(x_1,x_2,t)=\chi_0(x_2)e^{\iota k(x_1-ct)}\\ \psi(x_1,x_2,t)=\psi_0(x_2)e^{\iota k(x_1-ct)}\end{cases}, \tag{S15}$$

where $\iota=\sqrt{-1}$, k is the wavenumber, and c is the phase velocity. Inserting Eq. (S15) into Eqs. (S1) and (S11), we can get

$$\begin{cases}\chi=Ae^{-\xi kx_2}e^{\iota k(x_1-ct)}\\ \psi=[B_1\cosh(s_1kx_2)+B_2\sinh(s_1kx_2)+B_3\cosh(s_2kx_2)+B_4\sinh(s_2kx_2)]e^{\iota k(x_1-ct)}\end{cases}. \tag{S16}$$

The parameters s_1, s_2 and ξ are determined by

$$\gamma s^4-(2\beta-\rho c^2)s^2+(\alpha-\rho c^2)=0, \tag{S17}$$

and

$$\xi^2 - 1 = -c^2\rho^f/\nu. \tag{S18}$$

Substituting $\psi(x_1, x_2, t)$ and $\chi(x_1, x_2, t)$ in Eqs. (S13) and (S14) in terms of Eq. (S16) we get

$$M_{5\times5} \cdot [B_1, B_2, B_3, B_4, A]^{\mathrm{T}} = 0, \tag{S19}$$

where the nonzero components of $\mathbf{M}$ are

$$\begin{gathered}
M_{11} = s_1^2 + 1, M_{13} = s_2^2 + 1, \\
M_{22} = \gamma s_1(s_2^2 + 1), M_{24} = \gamma s_2(s_1^2 + 1), M_{25} = \iota\rho^f c^2, \\
M_{31} = 1, M_{33} = 1, M_{35} = -\iota\xi, \\
M_{41} = (s_1^2 + 1)\cosh(s_1 kh), M_{42} = (s_1^2 + 1)\sinh(s_1 kh), \\
M_{43} = (s_2^2 + 1)\cosh(s_2 kh), M_{44} = (s_2^2 + 1)\sinh(s_2 kh), \\
M_{51} = s_1(s_2^2 + 1)\sinh(s_1 kh), M_{52} = s_1(s_2^2 + 1)\cosh(s_1 kh), \\
M_{53} = s_2(s_1^2 + 1)\sinh(s_2 kh), M_{54} = s_2(s_1^2 + 1)\cosh(s_1 kh).
\end{gathered} \tag{S20}$$

In this derivation, we use the identity

$$2\beta - \rho c^2 = \gamma(s_1^2 + s_2^2), \tag{S21}$$

which can be obtained from Eq. (S17). The existence of nontrivial solution to Eq. (S12) requires

$$\det(\mathbf{M}_{5\times5}) = 0, \tag{S22}$$

which is the secular equation for the guided waves in the cornea.

Reference

1. M. Destrade and G. Saccomandi, Waves in nonlinear pre-stressed materials. *Springer Science & Business Media.* **495**, 1–26 (2007).
2. T. C. Gasser, R. W. Ogden, G. A. Holzapfel, Hyperelastic modelling of arterial layers with distributed collagen fibre orientations. *J. R. Soc. Interface*. **3**, 15–35 (2005).
3. G.-Y. Li, Q. He, R. Mangan, G. Xu, C. Mo, J. Luo, M. Destrade, Y. Cao, Guided waves in pre-stressed hyperelastic plates and tubes: Application to the ultrasound elastography of thin-walled soft materials. *J. Mech. Phys. Solids*. **102** (2017).
4. M. Otténio, M. Destrade, R. W. Ogden, Acoustic waves at the interface of a pre-stressed incompressible elastic solid and a viscous fluid. *Int. J. Non. Linear. Mech.* **42**, 310–320 (2007).

Supplementary Note 2. Phase velocities of *A*₀ and *S*₀ modes at high/low frequency regimes

When the wave frequency approaches to the infinity, the dispersion relations of A0 and S0 exhibit plateaus (Fig. S2A). The corresponding phase velocities are denoted by c_∞^{A0} and c_∞^{S0}, respectively. c_∞^{A0} is equivalent to the speed of the interfacial wave at the fluid-cornea interface (Scholte wave). c_∞^{S0} is equivalent to the speed of the surface wave at the free surface of the cornea (Rayleigh wave). $c_S = \sqrt{\alpha/\rho}$ is the bulk shear wave speed along the x_1 axis. In the absence of prestress (i.e., $\lambda = 1$), $c_S = \sqrt{\mu/\rho}$. The dependences of $c_\infty^{\mathrm{A0}}/c_S$ and $c_\infty^{\mathrm{S0}}/c_S$ on the material anisotropy $(2k_1/\mu)$ are shown in Fig. S2B. $c_\infty^{\mathrm{A0}}/c_S$ and $c_\infty^{\mathrm{S0}}/c_S$ increase slightly with $2k_1/\mu$ but are always smaller than 1. For isotropic material $(k_1/\mu = 0)$, we have $c_\infty^{\mathrm{A0}}/c_S \approx 0.839$, $c_\infty^{\mathrm{S0}}/c_S \approx 0.955$. These values close to 1 indicate that c_∞^{A0} and c_∞^{S0} are close to the bulk shear wave speed c_S, and therefore probing c_∞^{A0} and c_∞^{S0} gives us a good estimate of the shear modulus μ.

The phase velocity of S0 at zero frequency is nonzero and denoted by c_0^{S0}. It can be shown that $c_0^{\mathrm{S0}} = \sqrt{(2\beta + 2\gamma)/\rho}$. In the absence of prestress (see Eq. (S10)), we have $c_0^{\mathrm{S0}} = 2\sqrt{(\mu + k_1)/\rho}$ (Fig. S2B), from which we find that probing c_0^{S0} gives us the access to the anisotropic material parameter k_1. In fact, c_0^{S0} is determined by the plane-strain Young's modulus along the x_1 axis, which is the ratio between the uniaxial stress and the strain when the material is stretch along the x_1 axis while the out-of-plane direction (x_3) is completely constrained. We find that the plane-strain modulus E_1^* is given by $E_1^* = 4\mu + 4k_1$. For isotropic materials $(k_1/\mu = 0)$, E_1^* is four times the shear modulus, a well-known result for incompressible materials (Poisson's ratio is 0.5).

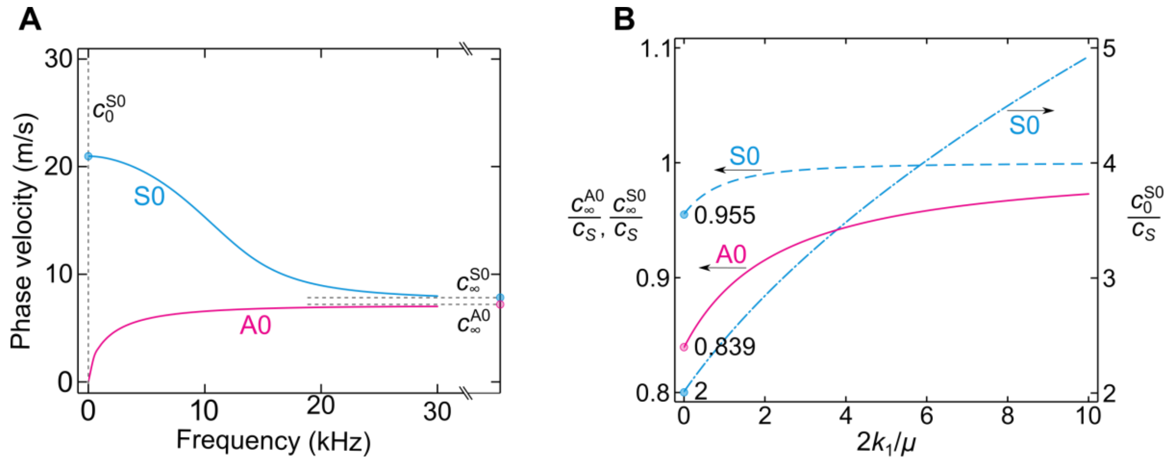

Fig. S2 (A) Phase velocities of A0 and S0 waves over frequency. c_∞^{A0} and c_∞^{S0} denote the phase velocities of the A0 and S0 waves at infinite frequency. c_0^{S0}, phase velocity of the S0 at zero frequency. (B) Dependences of c_∞^{A0}, c_∞^{S0}, and c_0^{S0} on the material anisotropy. For isotropic materials $(2k_1/\mu = 0)$, we obtain $c_\infty^{\mathrm{A0}}/c_S \approx 0.839$ (Scholte wave), $c_\infty^{\mathrm{S0}}/c_S \approx 0.955$ (Rayleigh wave), and $c_0^{\mathrm{S0}}/c_T = 2$.

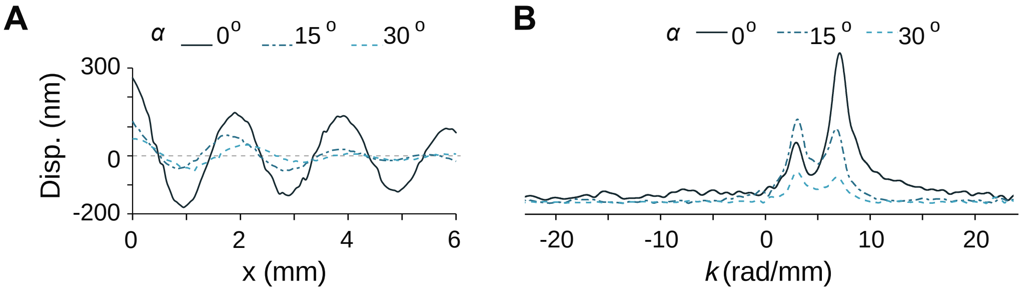

Fig. S3 Optimization of the probe tip angle α. (A) Total displacement amplitude at 4 kHz at different angles. (B) Wavenumber domain plot showing the amplitude ratio of the S0 and A0 waves at 12 kHz.

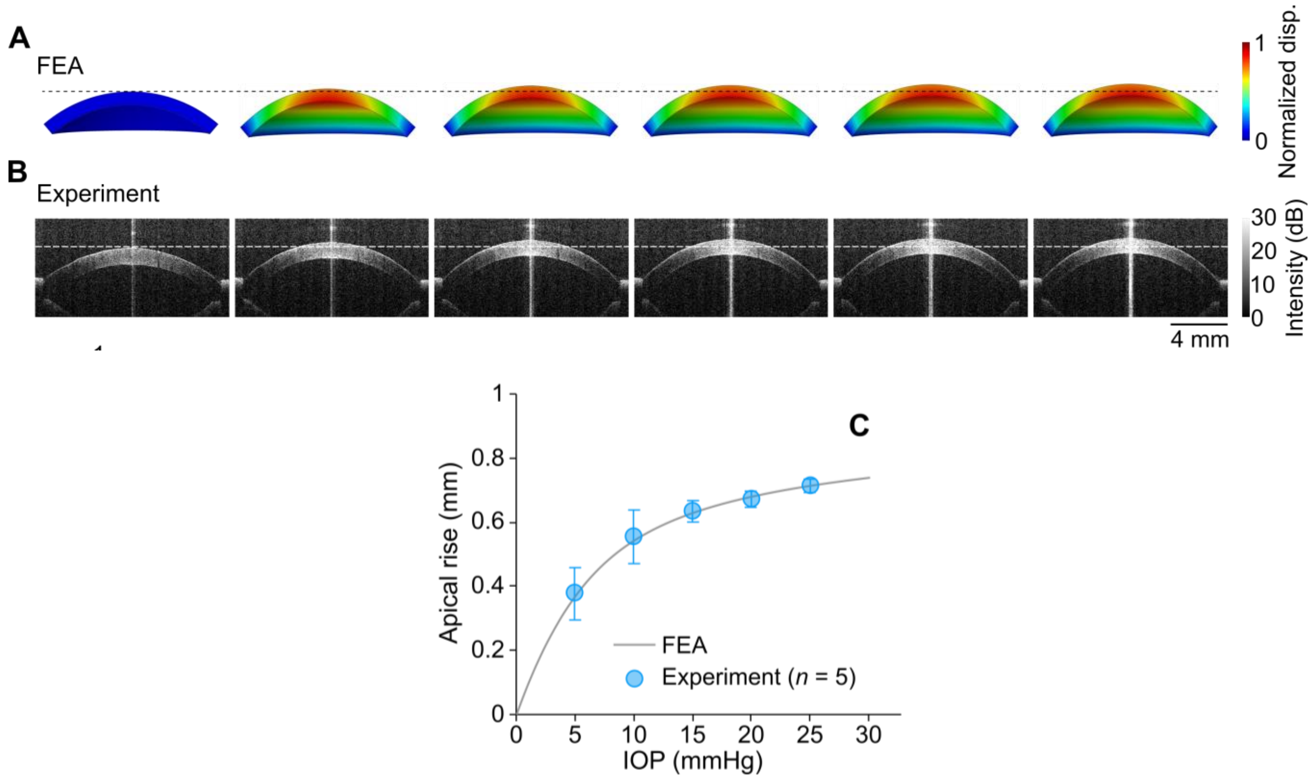

Fig. S4 Inflation test of the porcine cornea. (A) FEA to show deformation of the cornea. (B) OCT images of the inflated corneas as the intraocular pressure is increased. From left to right, IOP = 0, 5, 10, 15, 20, and 25 mmHg. (C) Comparison of the apical rise between FEA and experiment (N=5).
